# Differential Regulation of Adhesion and Phagocytosis of Resting and Activated Microglia by Dopamine

**DOI:** 10.3389/fncel.2018.00309

**Published:** 2018-09-11

**Authors:** Yang Fan, Zhilu Chen, Janak L. Pathak, Ana M. D. Carneiro, Chang Y. Chung

**Affiliations:** ^1^School of Pharmaceutical Science and Technology, Tianjin University, Tianjin, China; ^2^Department of Pharmacology, Vanderbilt University Medical Center, Vanderbilt University, Nashville, TN, United States

**Keywords:** microglia, dopamine, p38MAPK, paxillin, phagocytosis

## Abstract

Microglia, the immune competent cells of the central nervous system (CNS), normally exist in a resting state characterized by a ramified morphology with many processes, and become activated to amoeboid morphology in response to brain injury, infection, and a variety of neuroinflammatory stimuli. Many studies focused on how neurotransmitters affect microglia activation in pathophysiological circumstances. In this study, we tried to gain mechanistic insights on how dopamine (DA) released from neurons modulates cellular functions of resting and activated microglia. DA induced the reduction of the number of cellular processes, the increase of cell adhesion/spreading, and the increase of vimentin filaments in resting primary and BV_2_ microglia. In contrast to resting cells, DA downregulated the cell spreading and phagocytosis of microglia activated by LPS. DA also significantly downregulated ERK1/2 phosphorylation in activated microglia, but not in resting microglia. Downregulation of ERK1/2 by DA in activated microglia required receptor signaling. In contrast, we found a significant increase of p38MAPK activity by DA treatment in resting, but not in activated microglia. These latter effects required the uptake of DA through the high-affinity transporter but did not require receptor signaling. Activation of p38MAPK resulted in the increase of focal adhesion number via phosphorylation of paxillin at Ser^83^. These results indicate that DA might have a differential, depending upon the activation stage of microglia, impact on cellular functions such as adhesion and phagocytosis.

## Introduction

Microglia, immune competent cells of the central nervous system (CNS), normally exist in a resting state characterized by a ramified morphology and become activated into a motile ameboid form in response to brain injury, infection, and a variety of neuroinflammatory stimuli (Kreutzberg, 1996; Jacobowitz et al., 2012). They play a crucial role in recognition and phagocytic removal of degenerating neurons (Hickey, 2001; Streit, 2002). In neurological diseases and diverse brain insults, microglia promote the innate neuroinflammatory response responsible for brain repair (Kreutzberg, 1996; Streit et al., 2004). However, this inflammatory cascade can become pathogenic and contribute to the progression of disease processes. Microglia hyperactivation often leads to neurotoxicity and several neurodegenerative diseases accompanied by an excess proliferation of microglia (Gehrmann et al., 1995). Chronic activation of microglia may cause neuronal damage and prominent roles for microglia in neuroinflammation have also been suggested in chronic neurological diseases such as Alzheimer’s disease (AD), human immunodeficiency virus-1 (HIV-1) encephalopathy, prion diseases, and multiple sclerosis (MS) (Nelson et al., 2002; Streit, 2002; Streit, 2004; Lee et al., 2005).

Neurons are known to use different signals to control microglia: ‘Off’ signals keep microglia in their resting state and ‘On’ signals induce microglia activation (Biber et al., 2007). Microglia express several neurotransmitter receptors which modulate microglial functions. Activation of GABA(B) receptors and metabotropic purinergic receptors attenuated the release of interleukin-6 (IL-6) and microglia activation induced by lipopolysaccharide (LPS) (Boucsein et al., 2003; Kuhn et al., 2004). In contrast, activation of glutamate receptors enhanced the production of the tumor necrosis factor α(TNFα) (Noda et al., 2000). Many studies also reported that DA may affect several functions of innate immune system cells such as dendritic cells, macrophages, microglia, and neutrophils (Pinoli et al., 2017). DA decreases LPS-induced proliferation of human monocytes (Bergquist et al., 2000). It has been also shown that DA, acting on D1 receptor, can mitigate the proinflammatory response of LPS-activated macrophages and prevent systemic and neuro-inflammation *in vivo* (Yan et al., 2015). A previous study has reported a functional expression of D1 and D2 dopamine (DA) receptors in neonatal mouse microglia cultures and brain slices (Farber et al., 2005). Another study demonstrated that activated elderly microglia express DA receptors (Mastroeni et al., 2009). The presence of serotonin (5-HT) receptors on microglia were also confirmed. Amoeboid microglia respond to serotonin with a higher chemotactic response to ATP but with a decreased phagocytic activity (Krabbe et al., 2012). These reports clearly suggest that monoamine neurotransmitters can modulate microglia activation or behavior in normal and pathophysiological circumstances. However, most of these studies focused on how neurotransmitters affect the phagocytic behavior of activated microglia. Recently, emerging roles of resting or ramified microglia in several brain physiological functions including adult hippocampal neurogenesis, and cognitive and behavioral functions have been demonstrated. Microglia cells were shown to be highly active in the resting state, continually surveying their microenvironment with extremely motile processes and protrusions (Nimmerjahn et al., 2005). Ramified microglia have an important function in modulating hippocampal neurogenesis via phagocytosis of apoptotic cells during the 1st days of their life (Sierra et al., 2010). The cellular and molecular mechanisms underlying the regulation of resting microglia functions are largely unknown.

Under neuroinflammatory conditions, neurons release multiple neurotransmitters, at varying levels. For example, brain injury or emotional stress cause changes in DA release to the surroundings (Rougé-Pont et al., 1993; Pruessner et al., 2004). Thus, we tried to gain mechanistic insights how monoamine neurotransmitters released from neurons modulate cellular functions of resting and activated microglia. In this study, we demonstrated that uptake of DA through transporters cause a significant increase of p38 MAP kinase activity in resting, but not in activated microglia. Activation of the p38MAPK result in the increase of paxillin phosphorylation at Ser^83^, which might cause changes of cell shape via the increase of focal adhesions and vimentin filaments. DA, however, showed no significant effect on vimentin filaments and induced the decrease of phagocytosis in activated microglia. These results indicate that monoamine neurotransmitters might have a direct and significant impact on phagocytic activity and motility of microglia in different activation stage.

## Materials and Methods

### Cell Culture and Reagents

BV_2_ microglia cells were cultured in Dulbecco’s Modified Eagle’s Medium (DMEM) medium (Corning, Manassas, VA, United States) containing 10% fetal bovine serum (Biological Industries, Cromwell, CT, United States) and 100 U/mL penicillin/streptomycin (Solarbio, Beijing, China) in thermostatic incubator with 5% CO2. Primary microglial cells were prepared from brains of newborn pups of C57BL/6 mice (Huafukang Animal Center, Beijing, China). Briefly, decapitated heads were sterilized with ethanol and kept in ice-cold PBS. The scalp was cut open using fine scissors and brains were scooped out. Meninges were removed while brains were kept in the cold DMEM. The brain is then minced into small pieces using a pair of corneal scissors and tissues were dissociated into smaller pieces with repeated pipetting. Cells were collected by centrifuging at 400 rpm for 3 min, and the pellet was resuspended in DMEM with 10% fetal bovine serum and 100 U/mL penicillin/streptomycin. The cell suspension was added to poly-D-lysine coated flasks. Mixed glial cell cultures were incubated for 2 weeks, and microglia cells were detached by shaking the flasks on an orbital shaker at 200 rpm for 2 h at 37°C, and primary microglia cells were collected by centrifuging at 800 rpm for 3 min. Cells were resuspended in DMEM with 10% FBS and plated in poly-D-lysine (Sigma-Aldrich, Shanghai, China) coated plates. The purity of these cells was examined under a phase contrast microscope and fixed for staining with an Iba1 antibody (1:500) specific to microglia. All experiments using mice were in accordance with the Guide for the Care and Use of Laboratory Animals and were approved by the Research Ethics Committee, Tianjin Medical University.

DA and DA receptor antagonists [Haloperidol: D2, D3, D4 antagonist, Spiperone: D1, D2, D3, D4 antagonists, R-Bromo-alpha-Ergocryptine: D2, D3 antagonist, (+/-)-Sulpiride: D2 antagonist, Eticloprideon: D2 antagonist] were purchased from Sigma-Aldrich. Monoamine oxidase inhibitor, pargyline, was purchased from Sigma-Aldrich. Rasagiline and DA transporter blockers, Benztropine and Vanoxerine (GBR-12909), were purchased from MedChemExpress (Shanghai, China) and decynium-22, a blocker for plasma membrane monoamine transporter was from Sigma-Aldrich. A p38MAPK inhibitor, SB203580, was from Selleck Co. (Shanghai, China).

Antibodies against phospho-p44/p42 MAPK (pERK1/2), p44/p42 MAPK (ERK1/2), phospho-p38MAPK, p38MAPK, and vimentin were purchased from Cell Signaling Technology (Shanghai, China). Antibodies against phospho-paxillin (Ser-83) and paxillin were from ECM biosciences (Versailles, KY, United States). Antibody against Iba-1 was from Proteintech (Rosemont, IL, United States). We used 1:2000 dilutions of antibodies for immunoblots and 1:500 for immunofluorescence staining.

Transfection of BV_2_ and primary microglia cells was done with Viafect transfection agent (Promega Biological Products, Shanghai, China) according to the manufacturer’s protocol.

### Immunoblotting

Primary and BV_2_ microglia cells (80% confluent) were used to perform experiments. For activated microglia, cells were incubated with 100 ng/ml LPS for 6 h, which is a commonly used concentration for microglia activation in previous reports (Pruessner et al., 2004). Cells were then treated with 2 μM DA and incubated for various time. Cells were lysed with SDS-PAGE sample buffer containing protease inhibitor cocktail (Sigma-Aldrich) and phosphatase inhibitor (β-glycerophosphate). Lysates were denatured by heating at 95°C. Proteins were separated by SDS-PAGE and transferred onto PVDF membrane. The blots were blocked with 5% non-fat milk or 5% BSA in TTBS (Tris-buffered saline with 0.1% Tween-20) for 30 min and incubated overnight at 4°C with a primary antibody. Immunoblots were imaged with an Amersham Imager 600 (GE Healthcare), and bands were quantified using ImageJ software.

### Transfection and Fluorescence Microscopy

BV_2_ were grown on a flamed cover glass coated with poly-D-lysine at 50–60% confluence and transfected with Paxillin-GFP (2 μg) using 8 μl of ViaFect Transfection Reagent (Promega Biological Products, Shanghai, China) in OPTI-MEM (Thermo Fisher, Shanghai, China) for 12 h and cells were further incubated with DMEM with 10% FBS for 12 h. For primary microglia, cells were transfected in DMEM with 10% FBS for 24 h and further incubated for 12 h after medium change. Cells were then treated with 2 μM DA and inhibitors for 30 min, washed with PBS, and fixed with 4% fresh formaldehyde in PBS. Cells were rinsed three times (5 min each) with TBS-Tween, and the cover glass was mounted on slides. Cells were observed under a Nikon Ti fluorescence microscope (Tokyo, Japan) with 100× objective and images were taken with Qclick CCD camera (Qimaging, Surrey, Canada). The length of vimentin and area of cells were measured by using Image J software.

For phagocytosis assay, 1 μl of Alexa594-labeled latex beads (1 μm diameter; Molecular Probe, Shanghai, China) were added to the medium and incubated for 40 min. Cells were then fixed, washed, and permeabilized with 0.1% triton x-100. F-actin was stained with Alexa^488^-labeled phalloidin.

Primary astroglia cell mixture was grown on the coverglass coated with 0.1% poly-D- Lysine. Cells were treated with 2 μM DA and inhibitors for 30 min and fixed. Cells were then permeabilized with 0.2% Triton X-100 in PBS for 5 min, followed by incubation with blocking buffer (5% BSA, 0.1% Triton X-100 in PBS) for 30 min. Cells were incubated with the anti-paxillin antibody (mouse) and anti-Iba-1 antibody (rabbit) for overnight at 4°C with constant shaking, washed three times with TBS-T and incubated with fluorescence-conjugated (Alexa 488 and 594) secondary antibodies (Santa Cruz Biotechnology, United States) for 1 h. Cells were rinsed three times, mounted on slides, and observed under Nikon A1^+^ confocal microscope.

### RNA Isolation and Real-Time RT-PCR

Total RNA was isolated from BV_2_ cell cultures (80% confluent) by using an Eastep Super RNA isolation kit (Promega Biological Products, Shanghai, China) according to the manufacturer’s instructions. Total RNA concentrations were measured with a Nanodrop spectrophotometer (Thermo Scientific, Wilmington, DE, United States). cDNA synthesis was performed in a thermocycler GeneAmp^®^ PCR System 9700 PE (Applied Biosystems, Foster City, CA, United States), using a HiFi Script 1st strand cDNA synthesis kit (CW Biotech, China) with 1 μg of total RNA in 20 μl reaction. cDNA was diluted to 300 μl in DNase free water. Real-time PCR reactions for cDNA were performed using 5 μl cDNA and Ultra SYBR mixture with low ROX (CW Biotech, China) according to the manufacturer’s instructions in a LightCycler (Applied Biosystems, Quant studio 6 Flex real-time PCR system, San Francisco, CA, United States). Real-time PCR was used to assess expression of the dopamine transporter (*SLC6A3*), plasma membrane monoamine transporter (*SLC29A4*), organic cation transporter (*SLC22A1*) *1*, OCT2 (*SLC22A2*), and OCT3 (*SLC22A3*) genes. For quantitative real-time PCR, the values of relative target gene expression were normalized to *Hprt* housekeeping gene expression. We used *Hprt* as an internal housekeeping control since it provides the most consistent and stable *C*t value among β-Actin, GAPDH, and *Hprt*. The primers used for real-time PCR are listed in **Table 1**.

**Table 1:** Primers used in the real-time PCR assay.

Gene		Oligonucleotide Sequence	Amplicon Length (bp)
*Hprt*	Forward	5′ TGCTGACCTGCTGGATTACA 3′	118
	Reverse	5′ TATGTCCCCCGTTGACTGAT 3′	
*SLC6A3*	Forward	5′ TGGCCATGGTGCCCATTTAT 3′	122
	Reverse	5′ CACCTCCCCTCTGTCCACTA 3′	
*SLC29A4*	Forward	5′ CGGTCCCAAGCCTCGAGAA 3′	268
	Reverse	5′ TCTGTGAAAGTGGGGACAC 3′	
*SLC22A1*	Forward	5′ CTGGAGCACGTTGGAGAGTT 3′	96
	Reverse	5′ GATGCCCACGTAGATGGGAG 3′	
*SLC22A2*	Forward	5′ GAGTTCCCGCTGGTCGTATT 3′	294
	Reverse	5′ CAGGGGTAAGTGAGGTTGGG 3′	
*SLC22A3*	Forward	5′ GCCCGGAGCTCTCTTAATCC 3′	258
	Reverse	5′ CACAAGCCTGAGCAGAGTGA 3′	

### Data Analysis

All data was analyzed using Prism 6 software (GraphPad software). Experiments were analyzed using one-way ANOVA and Tukey corrections for multiple testing between categories (indicated in figure legends). The number of biological replicates (N) and statistical results are presented in the figure legends. The reported data for imaging studies originates from the initial analysis by the first experimenter. Data is presented with means ± standard error of the mean (SEM) shown as line and whiskers.

## Results

### Differential Regulation of Morphology and Cytoskeleton by DA in Resting and Activated Microglia

**FIGURE 1:**
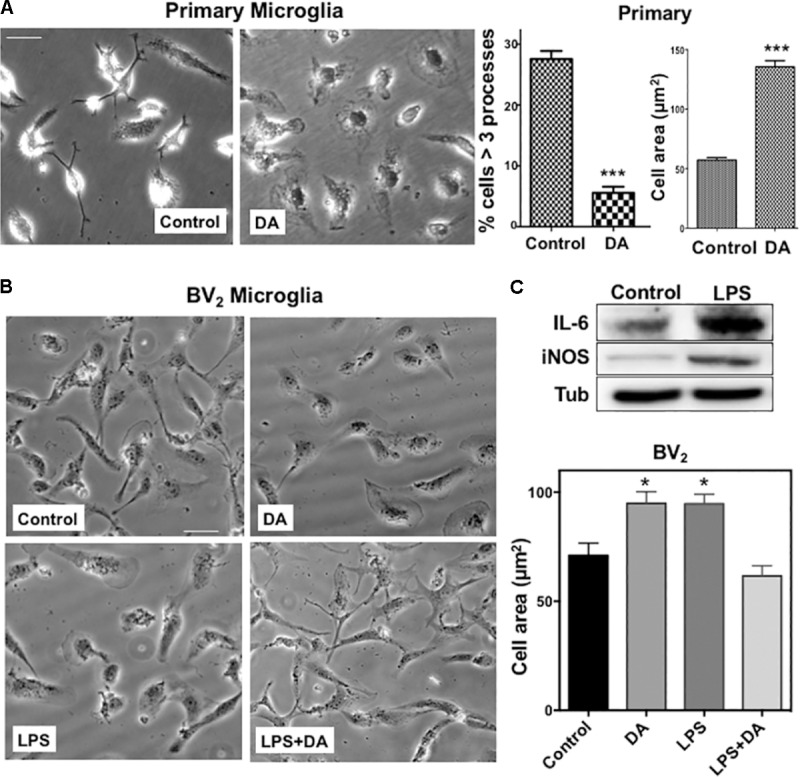
**(A)** Changes in the morphology of primary microglia upon DA treatment. Primary microglia cells showed small cell body and multiple processes. Upon DA (2 μM) treatment for 30 min, they acquired flattened morphology and area of cells were increased significantly, accompanied by a decreased number of cells having multiple processes (unpaired *t*-test: ^∗∗∗^*P* < 0.005). Values are means ± standard error of 15–20 cells. Right panels show the measurements of cellular process number and cell area. **(B)** BV_2_ microglia were stimulated with LPS and DA, and their morphology (left panels) and cell area (right panel) are shown (ANOVA: ^∗^*P* < 0.05 compared to vehicle control ^##^*P* < 0.05 compared to LPS). Scale bar = 20 μm. **(C)** Increased expression of IL-6 and iNOS upon LPS stimulation in BV_2_ cells.

**FIGURE 2:**
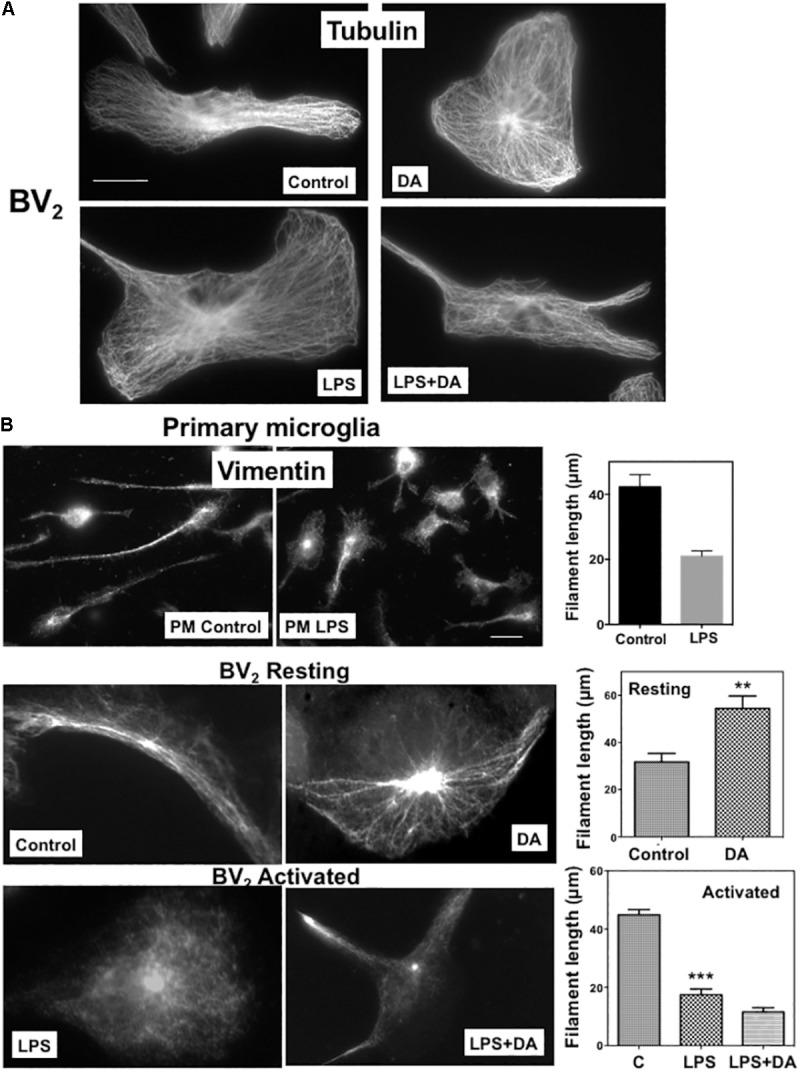
Changes in microglia cytoskeleton upon DA stimulation. **(A)** Microtubule organization shown by immunofluorescence staining with a β-tubulin antibody. Resting and activated BV_2_ microglia with or without DA (2 μM) treatment showed little changes in microtubule organization. Scale bar = 10 μm. **(B)** Changes of vimentin filaments in cells treated with DA for 30 min. The number and length of vimentin filaments in primary (PM) and BV_2_ microglia were visualized by immunofluorescence staining. Values are means ± standard error of 10–15 cells. DA (2 μM) induces the increase of number and length of vimentin filaments in resting BV_2_ cells. Loss of vimentin filaments and cell spreading upon the activation of microglia by LPS (100 ng/ml) were observed in both primary and BV_2_ microglia. DA could not rescue the loss of vimentin filaments in activated microglia. (ANOVA: ^∗∗∗^*P* < 0.005, ^∗∗^*P* < 0.01compare to control). Scale bar = 10 μm.

**FIGURE 3:**
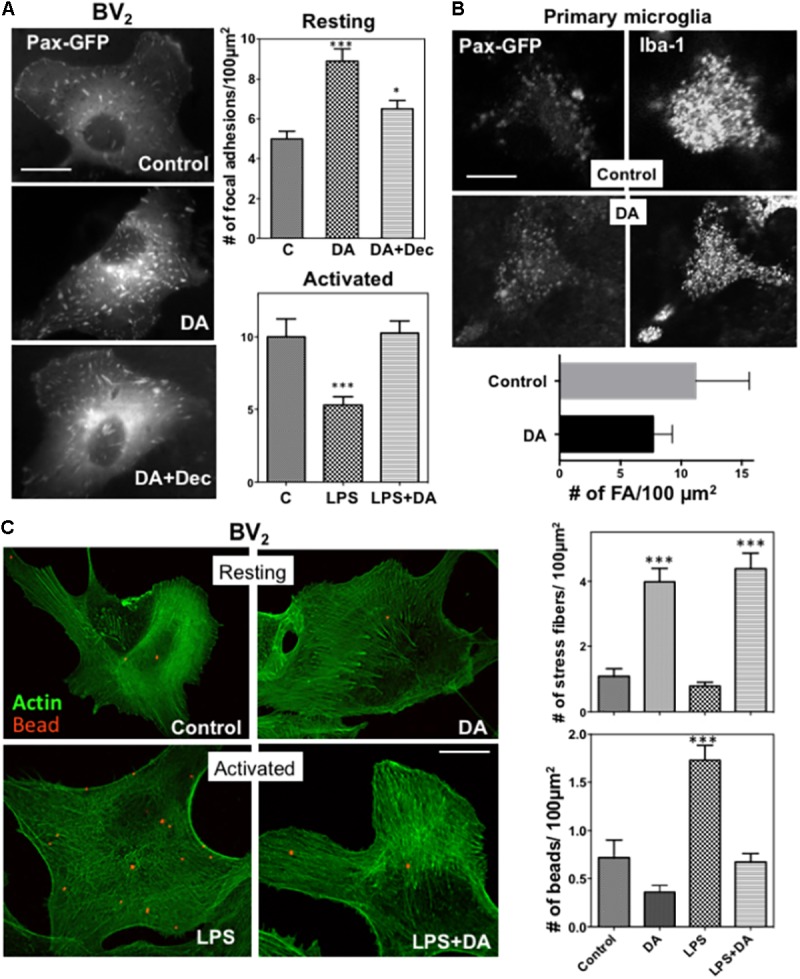
**(A)** Changes in focal adhesions in response to DA. BV_2_ cells were transfected with paxillin-GFP, and focal adhesions were imaged using fluorescence microscopy. The number of focal adhesions in resting cells was significantly increased in response to DA, which can be blocked by decynium (ANOVA: ^∗∗∗^*P* < 0.005 compared to control; ^∗^*P* < 0.05 compared to DA). Values are means ± standard error of 10–15 cells. Scale bar = 10 μm. **(B)** Primary astroglia mixture cultures were transfected with paxillin-GFP. Microglia cells were identified with Iba-1 staining and focal adhesions, visualized with paxillin-GFP, were imaged using confocal microscopy. Scale bar = 5 μm. **(C)** Phagocytic activity of resting and activated microglia was measured by examining the phagocytosis of beads labeled with Alexa 594 (1 μm diameter)) by cells for 30 min, followed by phalloidin staining. Treatment of DA results in a significant increase in the number of stress fibers in both resting and activated BV_2_ cells. A significant decrease of phagocytic activity was observed in activated microglia treated with DA. Values are means ± standard error of 10–15 cells. (ANOVA: ^∗∗∗^*P* < 0.005 compare to control ^###^*P* < 0.005 compare to LPS). Scale bar = 10 μm.

We examined how DA affects morphological changes of primary microglia. It has been reported that local DA levels can be increased up to the range of 100s of μM to the mM range in response to a sensory or pharmacological stimulus (Floresco et al., 2003). We used μM range of DA to avoid oxidative stress from high concentration of DA. In classical terms, “resting” microglia are naive cells that have not been exposed to inflammatory cytokines and they have branching processes and a relatively small cell body. About 30% of primary microglia cells have more than three processes, but the number of cellular processes was significantly reduced, and cell bodies appeared to be more flattened and spread in microglia treated with 2 μM DA for 30 min (**Figure 1A**), resulting in a significant increase in cell area. The DA-induced increase in cell spreading was also recapitulated in resting BV_2_ microglia cells (**Figure 1B**). Recent evidences indicated that microglia exhibit two functionally different activation states that are referred to as classical (pro-inflammatory, M1) and alternative activation (anti-inflammatory, M2) (Yang et al., 2017). Microglia exposed to LPS, a common stimulus that triggers a pro-inflammatory phenotype (M1 activated microglia) exhibited increased expression of IL-6 and iNOS, amoeboid morphology, and increased phagocytic activity (**Figures 1C**, **3C**). In contrast to resting cells, DA reduced the cell spreading of microglia activated by LPS (right panels). Prompted by changes in cell morphology and spreading, we examined the organization of microglial cytoskeletal by immunofluorescence microscopy. The number and organization of microtubules were not altered by DA either in resting or activated microglia (**Figure 2A**). We also examined changes in vimentin filaments upon DA treatment. Vimentin is a part of the intermediate filament system which plays an important physiological role in maintaining of cell shape and vimentin has been reported to function as a key controller for microglia activation (Jiang et al., 2013). Vimentin filaments were observed to run parallel to the longitudinal axis of the cell in primary and BV_2_ microglia (**Figure 2B**). Resting BV_2_ microglia treated with DA showed a significant increase in the number and length of vimentin filaments in different angles (**Figure 2B**), suggesting their contributions to the changes in shape/spreading. In contrast, activation of primary and BV_2_ microglia by LPS induced the loss of vimentin filaments. This loss was not further modified by DA in BV_2_ microglia.

Alterations in cell spreading of microglia also prompted us to examine changes in focal adhesions upon DA treatment, which were assessed by examining paxillin-GFP localization to focal adhesions in BV_2_ microglia. The number of focal adhesions were markedly increased in DA-treated resting BV_2_ cells (**Figure 3A**), consistent with its induction of cell spreading. To examine changes in focal adhesion in more physiological conditions, we transfected primary mixed astrocyte-microglia cultures with paxillin-GFP, and visualized focal adhesions using confocal microscopy. Consistent with results obtained from BV_2_ cells, significantly more focal adhesions were observed in microglia treated with DA (**Figure 3B**). The increase of focal adhesions upon DA stimulation was also observed in F-actin staining. Markedly increased number of stress fibers was observed in cells treated with DA, consistent with the increase of focal adhesions. LPS appeared to induce the significant loss of stress fibers (**Figure 3C**), and, unlike vimentin, DA can rescue the loss of stress fibers by LPS.

We examined how phagocytic activity of the microglia was affected by changes of the cytoskeleton and cell adhesion by examining engulfment of fluorescent beads. Cells were incubated with latex beads labeled with Alexa 594 (1 μm diameter)) for 30 min, then fixed, and further stained with Alexa 488-Phalloidin to visualize F-actin. Engulfed beads and F-actin were analyzed by confocal microscopy. To exclude beads bound to the surface of the cells, Bottoms of cells were focused using F-actin visualized with Alexa 488-phalloidin. Phagocytic activity of resting microglia was not significantly altered upon DA treatment (**Figure 3C**). Paralleling changes in cytoskeleton, activation of microglia by LPS resulted in enhanced phagocytic activity, which was normalized to resting levels by treatment of cells with DA (**Figure 3C**).

### Differential Regulation on the Activation of ERK1/2 and p38MAPK by DA in Resting and Activated Microglia

**FIGURE 4:**
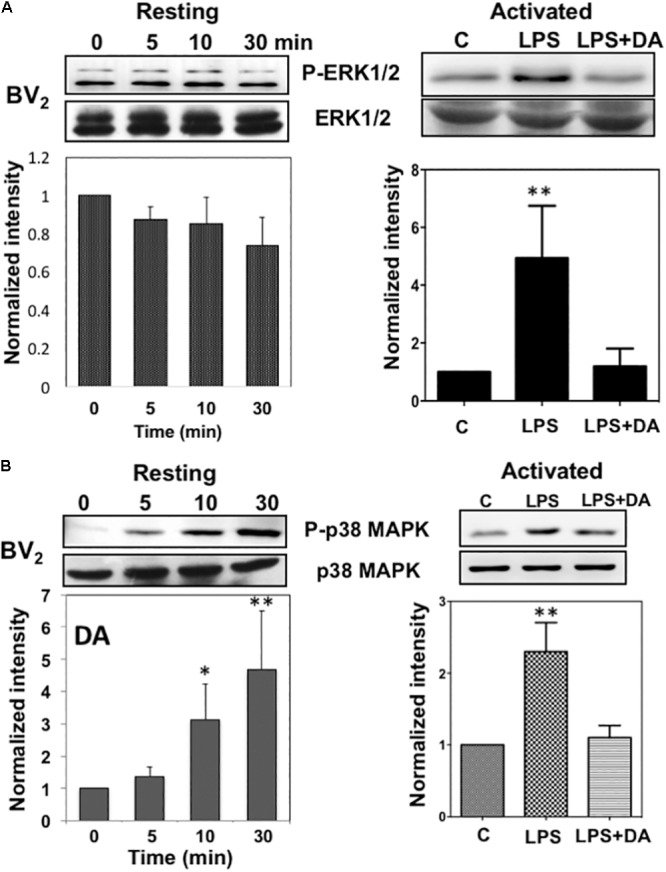
Immunoblot analysis of the activation of ERK1/2 and p38MAPK in resting and activated microglia. **(A)** Activation of ERK1/2 upon DA stimulation in resting and activated Bv2 microglia. Total cell lysates were collected at various time points (5, 10, and 30 min) after DA (2 μM) stimulation. The lysates were separated by SDS-PAGE, transferred onto PVDF membranes, and immunoblotted with the anti-phospho-ERK1/2 antibody. Immunoblots with anti-ERK1/2 antibody were used for loading control. ERK1/2 were measured as fold increase of the control. Values are means ± standard error of three or four independent experiments. **(B)** Activation of p38MAPK in resting, but not in activated microglia in response to 2 μM of DA. Both the resting and activated BV_2_ microglia were treated with 2 μM of DA for 30 min. Total cell lysates were separated by SDS-PAGE and immunoblotted with an anti-phospho-p38MAPK antibody. Values are means ± standard error of four independent experiments. (ANOVA: ^∗^*P* < 0.05, ^∗∗^*P* < 0.01 compared to control).

Activation of the mitogen-activated protein kinases, ERK1/2, has been reported in response to neurotransmitters (Troadec et al., 2002) and DA causes the phosphorylation of ERK1/2 via D1 receptor in neuroblastoma cells (Chen et al., 2004). We examined whether DA also can activate ERK1/2 in microglia cells by performing western blot analysis. Resting microglia were treated with 2 μM of DA for 5, 10, and 30 min and the activation of ERK1/2 was determined by immunoblot assays using phospho-ERK1/2 (Thr^202^/Tyr^204^) antibody (**Figure 4A**). ERK1/2 activation was not significantly altered from the control after the treatment of 2 μM DA after 30 min of DA treatment. Activation of microglia by LPS is known to activate ERK1/2 and p38MAPK. Treatment of LPS-activated microglia with 2 μM DA for 30 min reduced ERK1/2 phosphorylation to those observed in resting microglia, suggesting that DA can affect the activation status of microglia.

Lack of ERK1/2 activation by DA in resting microglia prompted us to examine the activation of anther mitogen-activated protein kinase, p38 MAP kinase (p38MAPK). p38MAPKs are a class of mitogen-activated protein kinases that are responsive to stress stimuli such as cytokines, ultraviolet irradiation, heat shock, as well as neurotransmitters (Cuadrado and Nebreda, 2010). The activation of p38MAPK was determined by immunoblot assays using phospho-p38MAPK (Thr180/Tyr182) antibody (**Figure 4B**). DA induced a significant increase of p38MAPK phosphorylation in resting microglia in a time-dependent manner. The peak of p38MAPK activation was observed at 30 min after DA treatment. Activation of microglia with LPS induced high levels of p38MAPK activation and DA treatment caused reductions in p38MAPK phosphorylation.

### Activation of p38MAPK by DA Cannot Be Blocked by Exposure to DA Receptor Antagonists

**FIGURE 5:**
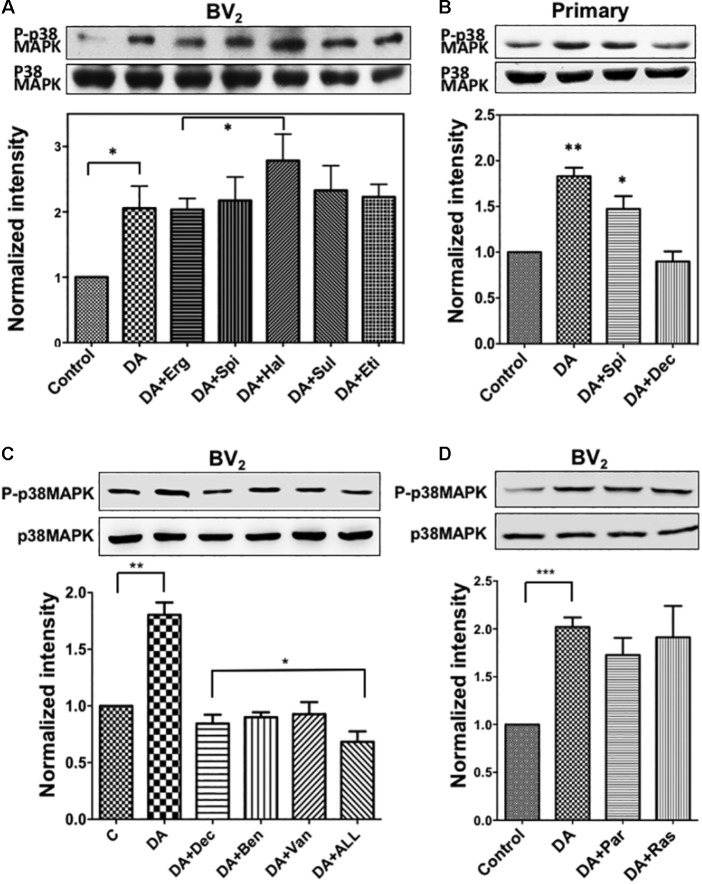
**(A)** Activation of p38MAPK by DA in resting microglia cannot be blocked with DA receptor antagonists. Resting BV_2_ microglia cells were pre-treated with various DA receptor antagonists (2 μM each, specificity of each antagonist was mentioned in section “Materials and Methods”) for 10 min and then treated with 2 μM DA for 30 min. Activation of p38MAPK was measured as fold increase to the control using immunoblotting with the anti-phospho-p38MAPK antibody. Values are means ± standard error of five independent experiments (ANOVA: ^∗^*P* < 0.05). **(B)** Activation of p38MAPK by DA in primary microglia. Cultured primary microglia cells from mouse brain were pre-treated with spiperone and decynium and treated with 2 μM DA for 30 min. Activation of p38MAPK was assessed by immunoblotting (ANOVA: ^∗^*P* < 0.05, ^∗∗^*P* < 0.01 compared to control). **(C)** p38MAPK activation by DA can be inhibited by selective DAT blockers (benztropine and vanoxerine; 2 μM each) and PMAT blocker (Decynium; 2 μM). Combination of DAT and PMAT blockers further inhibited the activation of p38MAPK (ANOVA: ^∗^*P* < 0.05, ^∗∗^*P* < 0.01). **(D)** Inhibitors of monoamine oxidase did not show any significant effect on p38MAPK activation by DA. Neither pargyline nor rasagiline (2 μM) showed a significant effect on p38MAPK activation by DA in resting microglia (ANOVA: ^∗∗∗^*P* < 0.005).

DA receptors compose a group of G protein-coupled receptors, and there are at least five subtypes; D_1_, D_2_, D_3_, D_4_, and D_5_ (Vallone et al., 2000). To examine if the activation of p38MAPK in resting microglia occurs via the stimulation of a DA receptor, we investigated the effects of different antagonists including Haloperidol, Spiperone, R-Bromo-alpha-Ergocryptine, (+/-)-Sulpiride, and Eticloprideon on DA-induced p38MAPK activation. Specificity of each antagonist was mentioned in the Materials section. We observed no alterations on DA-induced p38MAPK phosphorylation by antagonist treatment (**Figure 5A**), suggesting that p38MAPK might not be activated through DA receptors. Furthermore, haloperidol even induced a significant elevation of the p38MAPK phosphorylation up to 30% compared to the DA alone. To rule out a concern that BV_2_ cells might not behave like primary microglia, we repeated these experiments with primary microglia. DA can clearly activate p38MAPK and spiperone, an antagonist which could block different types of DA receptor in a wide range, did not show a significant inhibition, consistent with results from BV_2_ cells (**Figure 5B**).

### Differential Regulation of p38MAPK and ERK1/2 by DA

Many studies have found that high-affinity monoamine neurotransmitter transporters such as DA transporter (DAT), 5-HT transporter (SERT), and NE transporter (NET) are expressed on neuroglia cells (Solich et al., 2011). The plasma membrane monoamine transporter (PMAT) and organic cation transporter 3 (OCT3) are two other prominent low-affinity, high-capacity transporters for monoamine neurotransmitters (Duan and Wang, 2010). To examine the possibility that uptake of DA into the cell through transporter might cause the activation of p38MAPK, we used pharmacological inhibitors for these transporters; vanoxerine and benztropine (selective DAT blockers), and Decynium-22 (PMAT and OCT blocker). Both DAT and PMAT blockers showed significant inhibition on the activation of p38MAPK (**Figure 5C**), and maximum inhibition was seen by combining all three inhibitors, suggesting that DA uptake by both DAT and PMAT might be required for the subsequent activation of p38MAPK. Metabolism of uptake DA by monoamine oxidase (MAO) produces the reactive oxygen species (ROS) like H_2_O_2_ which induces the oxidative stress (Gluck et al., 2002). Oxidative stress might be one of the factors activating p38MAPK. Thus, we examined if pharmacological inhibitors for the monoamine oxidase, pargyline, and rasagiline, can inhibit p38MAPK activation. Neither pargyline nor rasagiline showed a significant effect on p38MAPK activation upon DA treatment (**Figure 5D**). This suggested the oxidative stress or MAO might not be a major route for the activation of p38MAPK.

**FIGURE 6:**
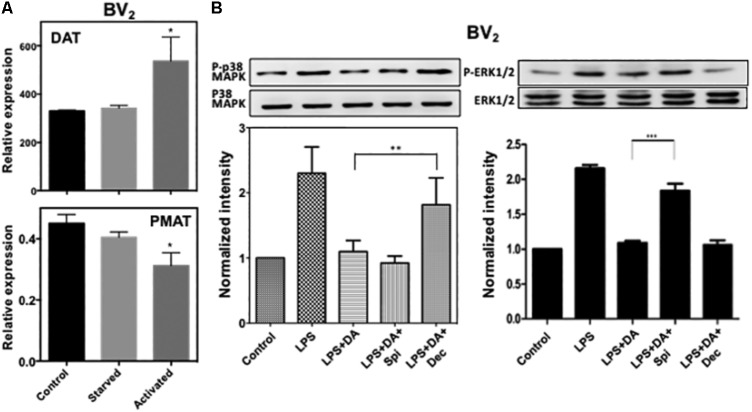
**(A)** Expression of DAT and PMAT in resting and activated microglia. Quantitative RT-PCR detected mRNA for DAT and PMAT in resting and activated BV_2_ microglia. The expression level of DAT increased significantly in activated microglia while PMAT expression decreased. (ANOVA: ^∗^*P* < 0.05 compared to control). **(B)** Activation of p38MAPK resulted from the activation of microglia by LPS can be blocked by decynium while activation of ERK1/2 can be blocked by spiperone (ANOVA: ^∗∗^*P* < 0.01, ^∗∗∗^*P* < 0.005).

To examine whether these transporters contributing to the uptake of DA and subsequent activation of p38MAPK are expressed in BV_2_ microglia cells, we used real-time RT-PCR. BV_2_ cells expressed DAT (mean ct value: 24.8) and PMAT (mean *C*t value: 33.4) genes, and their expression level appears to increase in serum-starved and activated microglia (**Figure 6A**). Expression of OCT1, OCT2, and OCT3 genes were not detected (*C*t value > 39) (data not shown).

We examined if the suppressive effect of DA on the activation of p38MAPK and ERK1/2 in microglia activated with LPS also requires DA uptake by transporters. Consistent to resting cells, decynium can alleviate the suppressive effect of DA on p38MAPK phosphorylation, while spiperone has little effects (**Figure 6**). These results suggest that suppression of p38MAPK activation by DA in activated microglia also requires reuptake of DA by transporters. In contrast, downregulation of ERK1/2 activation by DA in activated microglia appears to require DA receptors as spiperone alleviated the suppressive effect of DA on ERK1/2 activation in activated microglia. These results suggest that DA might regulate the activation of p38MAPK and ERK1/2 through different signaling routes.

### Activation of p38MAPK Results in the Phosphorylation of Ser^83^ of Paxillin

**FIGURE 7:**
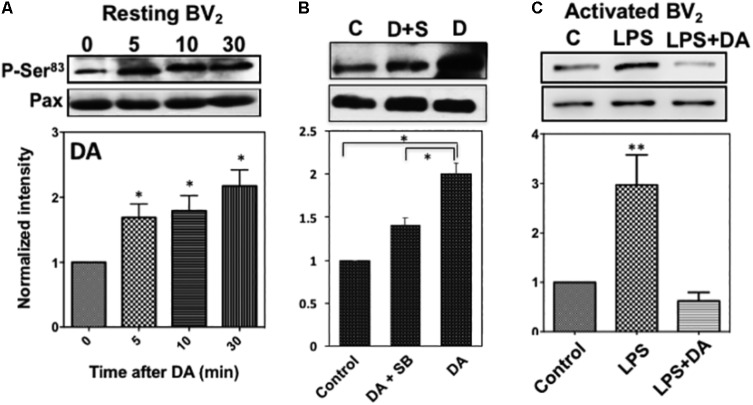
**(A)** Phosphorylation of paxillin at Ser^83^ via the activation of p38MAPK in resting microglia in response to DA. Paxillin phosphorylation was detected by immunoblots with the anti-phospho-paxillin Ser^83^ antibody. Immunoblots with anti-paxillin antibody were used as a loading control. Representative images of western blots for the phosphorylation of paxillin at Ser^83^ are shown. Values are means ± standard error of four independent experiments (ANOVA: ^∗^*P* < 0.05). **(B)** Inhibition of Ser^83^ phosphorylation by the p38MAPK inhibitor. Resting Bv2 microglia cells were pre-treated with 0.5 μM SB203580 (p38MAPK inhibitor) for 10 min and then 2 μM DA for 30 min. Values are means ± standard error of three independent experiments (ANOVA: ^∗^*P* < 0.05). **(C)** DA downregulates paxillin phosphorylation at Ser^83^ in activated microglia (ANOVA: ^∗∗^*P* < 0.01).

To understand how the activation of p38MAPK affects resting microglia functions, paxillin phosphorylation was examined since paxillin has been reported to be a major substrate for p38MAPK and ERK1/2 at Ser^83^ residue (Huang et al., 2004). Paxillin, a focal adhesion adaptor protein, is known to be involved in focal adhesion dynamics, cell migration, and phagocytosis (Tsubouchi et al., 2002; Webb et al., 2004; Gitik et al., 2014). Studies have shown that paxillin interacts with many proteins and these interactions result in changes in the organization of the actin cytoskeleton, which are necessary for controlling phagocytosis and cell motility (Turner, 2000; Duran et al., 2009). It has been reported that levels of phagocytosis, paxillin activation, and cofilin activation positively correlated with one another (Gitik et al., 2014). DA induced a significant increase of the paxillin Ser^83^ phosphorylation in resting microglia cells in a time-dependent manner (**Figure 7A**), indicating a positive correlation between p38MAPK activation and Ser83 phosphorylation of paxillin in resting microglia. A specific p38MAPK inhibitor, SB203580, significantly suppressed the level of phosphorylation on Ser^83^ (**Figure 7B**), confirming that the activation of p38MAPK results in the phosphorylation of Ser^83^. However, we can’t rule out the possibility that both ERK1/2 and p38MAPK are responsible for the phosphorylation of paxillin in activated microglia. Activation of microglia by LPS expectedly induced the phosphorylation of paxillin at Ser^83^, which can be down-regulated by DA (**Figure 7C**).

## Discussion

Microglia serves as the first defense of CNS and express a variety of neurotransmitter receptors so that neurotransmitter might have a significant influence on microglia functions (Pocock and Kettenmann, 2007). Brain injury or emotional stress cause changes in DA or 5-HT release to the surroundings (Kawahara et al., 1993; Chen et al., 2015). Microglia presumably exhibit differential responses to DA or 5-HT depending on their activation status. In this study, we described novel mechanisms for differential modulation of microglia function in resting and activated stage by DA. ERK1/2 activation has been shown as a key player for microglia response to different stimuli. LPS, a potent activator of microglia, can induce the activation of JNK1/2 and ERK1/2 signaling in microglia (Liu et al., 2014), resulting in the production of nitric oxide (NO) and the release of pro-inflammatory cytokines such as TNF-α and IL-1β (Zhao et al., 2014). We examined the differential responses of microglia in different activation stages (resting vs. activated) to DA. DA did not show a significant effect on ERK1/2 activation in resting microglia. In contrast, a significant increase of the activity of p38MAPK was observed in resting microglia upon DA treatment. Microglia have been shown to express a few DA receptors (Farber et al., 2005). It has been reported that dopamine D4 receptor antagonist can suppress microglial activation and delay the progression of amyotrophic lateral sclerosis (ALS) (Tanaka et al., 2008). DA, via D2 receptor, inhibits the pro-inflammatory response in resting state but both D1 and D2 agonists inhibited the proinflammatory responses in activated microglia (Dominguez-Meijide et al., 2017), indicating changes in the expression of DA receptor subtypes upon microglia activation. Our results demonstrated that none of the DA receptor antagonists inhibited the activation of p38MAPK upon DA stimulation in resting microglia. Furthermore, the activation of p38MAPK peaked after 15 min of DA treatment, which is significantly slower than receptor-mediated activation. These results indicate that DA receptor might not be the main route for p38MAPK activation. High affinity and specific transporters for DA, NE, or 5-HT constitute to reuptake of extracellular neurotransmitters (Bottalico et al., 2004). Additional monoamine transporters with low affinity and high capacity (OCT1, OCT2, OCT3, and PMAT) also play a key role in the uptake of monoamines from extracellular compartments (Bottalico et al., 2007; Zhou et al., 2007). Our results showed that both DAT and PMAT are expressed in microglia and blockers of DAT and PMAT can inhibit the activation of p38MAPK by DA in resting microglia. Receptor-independent activation of p38MAPK by DA has also been reported in rat C6 glioma cells (Luo et al., 1999).

It has been reported that metabolism of DA in the cell produces reactive oxygen species (ROS) and quinone species through autoxidation and enzyme-catalyzed reaction (Meiser et al., 2013). Monoamine oxidase (MAO), an enzyme bound to the outer membrane of mitochondria, catalyze the oxidation of monoamines and can generate H_2_O_2_ (Gluck et al., 2002). Due to its nature of being permeable through membrane and being relatively inert compared to other ROS, H_2_O_2_ has been suggested to be an intracellular and intercellular messengers (Avshalumov et al., 2004). It has been reported that DA metabolism by MAO followed by DA reuptake generates H_2_O_2_ which induces ERK1/2 activation and mitogenesis in renal epithelial cells (Vindis et al., 2001). In this study, however, the monoamine oxidase inhibitors did not show any significant changes in the p38MAPK activation, implying the presence of another signaling pathway through which DA activates p38MAPK.

Activated microglia via LPS treatment apparently induced high levels of ERK1/2 and p38MAPK activation, and DA downregulates the activation of both kinases. Many studies indicated that p38MAPK could be activated via various stimuli during microglia activation. In a streptozotocin-induced diabetes rat model, microglia activation in the spinal dorsal horn was mediated, in part, by phosphorylation of p-38 MAPK, which results in the persistent mechanical allodynia (Cheng et al., 2014). Another study showed that LPS-induced microglia activation results in the phosphorylation of p38MAPK, playing an important role in regulating immune responses to infection via modulation of NF-κB and AP-1 activities (Jeong et al., 2014). Downregulation of ERK1/2 and p38MAPK by DA could attenuate the activation of microglia, making a significant impact on the progression of neuroinflammation.

The function of microglia is closely associated with their motility and phagocytic activity. Changes in cytoskeleton generate the mechanical forces that drive phagocytosis. Our study is the first demonstration of the differential role of DA in the regulation of adhesion and phagocytosis of resting and activated microglia. Our study demonstrated that DA could enhance adhesion strength via the increase of focal adhesions, stress fibers, and vimentin filaments of resting microglia via the activation of p38MAPK and phosphorylation of Ser^83^ of paxillin, resulting in the decrease of cellular processes and cell spreading. Even though DA also appears to induce the increase of focal adhesion and stress fibers in activated microglia, cell spreading was significantly down-regulated in activated microglia. Furthermore, DA exhibits an inhibitory effect on phagocytic activity of activated microglia presumably via the downregulation of ERK1/2 and p38MAPK activity and phosphorylation of Ser^83^ of paxillin. Many reports indicated that the activation of the p38MAPK is linked with the motility and phagocytosis of activated microglia. Neuropeptide Y (NPY) inhibits microglia motility stimulated by LPS via the inhibition of p38MAPK phosphorylation, and p38MAPK is required for the phagocytosis of injured neurons by activated microglia (Tanaka et al., 2009; Ferreira et al., 2012; Katayama et al., 2012). As DA apparently showed the differential effect on the phosphorylation of Paxillin Ser^83^ in different stages of microglia, it is possible that role of p38MAPK in the regulation of phagocytosis might be different in different stages of microglia activation. Vimentin might play a key role in differential regulation of cell morphology and phagocytosis in resting and activated microglia. It has been demonstrated that the interaction of disassembled vimentin with phosphorylated ERK1/2 and p38MAPKs could mediate CCL2 production in mast cells upon activation (Toda et al., 2012). In this study, we demonstrated that DA significantly enhances the assembly of vimentin in resting microglia while has little effect on disassembled vimentin in activated microglia. The interaction of vimentin with ERK1/2 and p38MAPK might contribute to localizing the activity of these kinases toward various substrates including paxillin. Paxillin and cofilin are major components in controlling cytoskeleton function (Tsubouchi et al., 2002; Webb et al., 2004; Gitik et al., 2014). Paxillin is a known substrate for p38MAPK (Huang et al., 2004) and phosphorylations of paxillin play a key role in the regulation of microglia chemotaxis (Lee et al., 2012). It was reported that the phosphorylation of paxillin at Ser^83^ is required for adhesion disassembly. Phosphorylation of paxillin at Ser^83^ presumably result in a faster turnover rate of focal adhesions, resulting in higher number of adhesions as also shown in PC12 cells (Huang et al., 2004). Phosphorylation at Tyr^118^ has been shown to be positively correlated with the higher phagocytic activity of microglia (Gitik et al., 2014). Thus, phosphorylation of paxillin via the activation of p38MAPK might have a profound effect on the regulation of cytoskeleton and phagocytosis of microglia. Our results suggest that monoamine neurotransmitters might have a direct and significant impact on phagocytic activity and motility of microglia in different activation stage, making a significant impact on the progression of neuroinflammation.

Microglia hyperactivation often leads to neurotoxicity and several neurodegenerative diseases such as Parkinson’s disease (PD). PD is a neurodegenerative disorder characterized by the loss of dopaminergic neurons of the substantia nigra pars compacta and the depletion of DA in the striatum. Many studies using a variety of techniques demonstrated that reductions in DA content and uptake of DA in the brains of PD patients (Leenders et al., 1990; Chinaglia et al., 1992). Persistent microglial activation and higher phagocytic activity in the substantia nigra of the brain of PD patients has been reported (Barcia et al., 2013), suggesting that phagocytic characteristic of activated microglia may contribute to the degeneration of dopaminergic neurons. Downregulation of ERK1/2, cell spreading, and phagocytosis by DA in activated microglia are consistent with the role of DA attenuating the phagocytic characteristic of microglia in the progress of PD.

## Author Contributions

YF, ZC, JP, and CC performed the experiments and analyzed the data. YF, AC, and CC conceived the experiments, analyzed the data, and wrote the paper.

## Conflict of Interest Statement

The authors declare that the research was conducted in the absence of any commercial or financial relationships that could be construed as a potential conflict of interest.

## References

[B1] Avshalumov M. V. MacGregor D. G. Sehgal L. M. Rice M. E. (2004). The glial antioxidant network and neuronal ascorbate: protective yet permissive for H(2)O(2) signaling. *Neuron Glia Biol.* 1 365–376. 10.1017/S1740925X05000311 18292802 PMC2249559

[B2] Barcia C. Ros C. M. Ros-Bernal F. Gómez A. Annese V. Carrillo-de Sauvage M. A. (2013). Persistent phagocytic characteristics of microglia in the substantia nigra of long-term Parkinsonian macaques. *J. Neuroimmunol.* 261 60–66. 10.1016/j.jneuroim.2013.05.001 23759319

[B3] Bergquist J. Ohlsson B. Tarkowski A. (2000). Nuclear factor-kappa B is involved in the catecholaminergic suppression of immunocompetent cells. *Ann. N. Y. Acad. Sci.* 917 281–289. 10.1111/j.1749-6632.2000.tb05394.x 11268354

[B4] Biber K. Neumann H. Inoue K. Boddeke H. W. (2007). Neuronal ’On’ and ’Off’ signals control microglia. *Trends Neurosci.* 30 596–602. 10.1016/j.tins.2007.08.007 17950926

[B5] Bottalico B. Larsson I. Brodszki J. Hernandez-Andrade E. Casslén B. Marsál K. (2004). Norepinephrine transporter (NET), serotonin transporter (SERT), vesicular monoamine transporter (VMAT2) and organic cation transporters (OCT1, 2 and EMT) in human placenta from pre-eclamptic and normotensive pregnancies. *Placenta* 25 518–529. 10.1016/j.placenta.2003.10.017 15135235

[B6] Bottalico B. Noskova V. Pilka R. Larsson I. Domanski H. Casslén B. (2007). The organic cation transporters (OCT1, OCT2, EMT) and the plasma membrane monoamine transporter (PMAT) show differential distribution and cyclic expression pattern in human endometrium and early pregnancy decidua. *Mol. Reprod. Dev.* 74 1303–1311. 10.1002/mrd.20697 17393420

[B7] Boucsein C. Zacharias R. Färber K. Pavlovic S. Hanisch U. K. Kettenmann H. (2003). Purinergic receptors on microglial cells: functional expression in acute brain slices and modulation of microglial activation in vitro. *Eur. J. Neurosci.* 17 2267–2276. 10.1046/j.1460-9568.2003.02663.x 12814360

[B8] Chen J. Rusnak M. Luedtke R. R. Sidhu A. (2004). D1 dopamine receptor mediates dopamine-induced cytotoxicity via the ERK signal cascade. *J. Biol. Chem.* 279 39317–39330. 10.1074/jbc.M403891200 15247297

[B9] Chen Y. H. Huang E. Y. Kuo T. T. Ma H. I. Hoffer B. J. Tsui P. F. (2015). Dopamine release impairment in striatum after different levels of cerebral cortical fluid percussion injury. *Cell Transplant.* 24 2113–2128. 10.3727/096368914X683584 25198499

[B10] Cheng K. I. Wang H. C. Chuang Y. T. Chou C. W. Tu H. P. Yu Y. C. (2014). Persistent mechanical allodynia positively correlates with an increase in activated microglia and increased P-p38 mitogen-activated protein kinase activation in streptozotocin-induced diabetic rats. *Eur. J. Pain* 18 162–173. 10.1002/j.1532-2149.2013.00356.x 23868758

[B11] Chinaglia G. Alvarez F. J. Probst A. Palacios J. M. (1992). Mesostriatal and mesolimbic dopamine uptake binding sites are reduced in Parkinson’s disease and progressive supranuclear palsy: a quantitative autoradiographic study using [3H]mazindol. *Neuroscience* 49 317–327. 10.1016/0306-4522(92)90099-N 1436470

[B12] Cuadrado A. Nebreda A. R. (2010). Mechanisms and functions of p38 MAPK signalling. *Biochem. J.* 429 403–417. 10.1042/BJ20100323 20626350

[B13] Dominguez-Meijide A. Rodriguez-Perez A. I. Diaz-Ruiz C. Guerra M. J. Labandeira-Garcia J. L. (2017). Dopamine modulates astroglial and microglial activity via glial renin-angiotensin system in cultures. *Brain Behav. Immun.* 62 277–290. 10.1016/j.bbi.2017.02.013 28232171

[B14] Duan H. C. Wang J. (2010). Selective transport of monoamine neurotransmitters by human plasma membrane monoamine transporter and organic cation transporter 3. *J. Pharmacol. Exp. Ther.* 335 743–753. 10.1124/jpet.110.170142 20858707 PMC2993547

[B15] Duran M. B. Rahman A. Colten M. Brazill D. (2009). Dictyostelium discoideum paxillin regulates actin-based processes. *Protist* 160 221–232. 10.1016/j.protis.2008.09.005 19213599 PMC2743336

[B16] Farber K. Pannasch U. Kettenmann H. (2005). Dopamine and noradrenaline control distinct functions in rodent microglial cells. *Mol. Cell. Neurosci.* 29 128–138. 10.1016/j.mcn.2005.01.003 15866053

[B17] Ferreira R. Santos T. Cortes L. Cochaud S. Agasse F. Silva A. P. (2012). Neuropeptide Y inhibits interleukin-1 beta-induced microglia motility. *J. Neurochem.* 120 93–105. 10.1111/j.1471-4159.2011.07541.x 22007767

[B18] Floresco S. B. West A. R. Ash B. Moore H. Grace A. A. (2003). Afferent modulation of dopamine neuron firing differentially regulates tonic and phasic dopamine transmission. *Nat. Neurosci.* 6 968–973. 10.1038/nn1103 12897785

[B19] Gehrmann J. Matsumoto Y. Kreutzberg G. W. (1995). Microglia: intrinsic immuneffector cell of the brain. *Brain Res. Brain Res. Rev.* 20 269–287. 10.1016/0165-0173(94)00015-H 7550361

[B20] Gitik M. Kleinhaus R. Hadas S. Reichert F. Rotshenker S. (2014). Phagocytic receptors activate and immune inhibitory receptor SIRP alpha inhibits phagocytosis through paxillin and cofilin. *Front. Cell. Neurosci.* 8:104. 10.3389/fncel.2014.00104 24795566 PMC3997012

[B21] Gluck M. Ehrhart J. Jayatilleke E. Zeevalk G. D. (2002). Inhibition of brain mitochondrial respiration by dopamine: involvement of H2O2 and hydroxyl radicals but not glutathione-protein-mixed disulfides. *J. Neurochem.* 82 66–74. 10.1046/j.1471-4159.2002.00938.x 12091466

[B22] Hickey W. F. (2001). Basic principles of immunological surveillance of the normal central nervous system. *Glia* 36 118–124. 10.1002/glia.1101 11596120

[B23] Huang C. Borchers C. H. Schaller M. D. Jacobson K. (2004). Phosphorylation of paxillin by p38MAPK is involved in the neurite extension of PC-12 cells. *J. Cell Biol.* 164 593–602. 10.1083/jcb.200307081 14970194 PMC2171993

[B24] Jacobowitz D. M. Cole J. T. McDaniel D. P. Pollard H. B. Watson W. D. (2012). Microglia activation along the corticospinal tract following traumatic brain injury in the rat: a neuroanatomical study. *Brain Res.* 1465 80–89. 10.1016/j.brainres.2012.05.008 22617376

[B25] Jeong Y. H. Kim Y. Song H. Chung Y. S. Park S. B. Kim H. S. (2014). Anti-inflammatory effects of alpha-galactosylceramide analogs in activated microglia: involvement of the p38 MAPK signaling pathway. *PLoS One* 9:e87030. 10.1371/journal.pone.0087030 24523867 PMC3921125

[B26] Jiang X. Ni Y. Liu T. Zhang M. Ren H. Xu G. (2013). Inhibition of LPS-induced retinal microglia activation by naloxone does not prevent photoreceptor death. *Inflammation* 36 42–52. 10.1007/s10753-012-9518-6 22869199

[B27] Katayama T. Kobayashi H. Okamura T. Yamasaki-Katayama Y. Kibayashi T. Kimura H. (2012). Accumulating microglia phagocytose injured neurons in hippocampal slice cultures: involvement of p38 MAP kinase. *PLoS One* 7:e40813. 10.1371/journal.pone.0040813 22815830 PMC3398896

[B28] Kawahara H. Yoshida M. Yokoo H. Nishi M. Tanaka M. (1993). Psychological stress increases serotonin release in the rat amygdala and prefrontal cortex assessed by in vivo microdialysis. *Neurosci. Lett.* 162 81–84. 10.1016/0304-3940(93)90565-3 8121642

[B29] Krabbe G. Matyash V. Pannasch U. Mamer L. Boddeke H. W. Kettenmann H. (2012). Activation of serotonin receptors promotes microglial injury-induced motility but attenuates phagocytic activity. *Brain Behav. Immun.* 26 419–428. 10.1016/j.bbi.2011.12.002 22198120

[B30] Kreutzberg G. W. (1996). Microglia: a sensor for pathological events in the CNS. *Trends Neurosci.* 19 312–318. 10.1016/0166-2236(96)10049-7 8843599

[B31] Kuhn S. A. van Landeghem F. K. Zacharias R. Färber K. Rappert A. Pavlovic S. (2004). Microglia express GABA(B) receptors to modulate interleukin release. *Mol. Cell. Neurosci.* 25 312–322. 10.1016/j.mcn.2003.10.023 15019947

[B32] Lee D. Y. Oh Y. J. Jin B. K. (2005). Thrombin-activated microglia contribute to death of dopaminergic neurons in rat mesencephalic cultures: dual roles of mitogen-activated protein kinase signaling pathways. *Glia* 51 98–110. 10.1002/glia.20190 15789435

[B33] Lee S. H. Hollingsworth R. Kwon H. Y. Lee N. Chung C. Y. (2012). beta-arrestin 2-dependent activation of ERK1/2 is required for ADP-induced paxillin phosphorylation at Ser(83) and microglia chemotaxis. *Glia* 60 1366–1377. 10.1002/glia.22355 22638989 PMC3984973

[B34] Leenders K. L. Salmon E. P. Tyrrell P. Perani D. Brooks D. J. Sager H. (1990). The nigrostriatal dopaminergic system assessed in vivo by positron emission tomography in healthy volunteer subjects and patients with Parkinson’s disease. *Arch. Neurol.* 47 1290–1298. 10.1001/archneur.1990.00530120034007 2123623

[B35] Liu R. P. Zou M. Wang J. Y. Zhu J. J. Lai J. M. Zhou L. L. (2014). Paroxetine ameliorates lipopolysaccharide-induced microglia activation via differential regulation of MAPK signaling. *J. Neuroinflamm.* 11:47. 10.1186/1742-2094-11-47 24618100 PMC3995780

[B36] Luo Y. Kokkonen G. C. Hattori A. Chrest F. J. Roth G. S. (1999). Dopamine stimulates redox-tyrosine kinase signaling and p38 MAPK in activation of astrocytic C6-D2L cells. *Brain Res.* 850 21–38. 10.1016/S0006-8993(99)02021-1 10629745

[B37] Mastroeni D. Grover A. Leonard B. Joyce J. N. Coleman P. D. Kozik B. (2009). Microglial responses to dopamine in a cell culture model of Parkinson’s disease. *Neurobiol. Aging* 30 1805–1817. 10.1016/j.neurobiolaging.2008.01.001 18325635 PMC2762863

[B38] Meiser J. Weindl D. Hiller K. (2013). Complexity of dopamine metabolism. *Cell Commun. Signal.* 11:34. 10.1186/1478-811X-11-34 23683503 PMC3693914

[B39] Nelson P. T. Soma L. A. Lavi E. (2002). Microglia in diseases of the central nervous system. *Ann. Med.* 34 491–500. 10.1080/078538902321117698 12553488

[B40] Nimmerjahn A. Kirchhoff F. Helmchen F. (2005). Resting microglial cells are highly dynamic surveillants of brain parenchyma in vivo. *Science* 308 1314–1318. 10.1126/science.1110647 15831717

[B41] Noda M. Nakanishi H. Nabekura J. Akaike N. (2000). AMPA-kainate subtypes of glutamate receptor in rat cerebral microglia. *J. Neurosci.* 20 251–258. 10.1523/JNEUROSCI.20-01-00251.2000 10627602 PMC6774119

[B42] Pinoli M. Marino F. Cosentino M. (2017). Dopaminergic regulation of innate immunity: a review. *J. Neuroimmune Pharmacol.* 12 602–623. 10.1007/s11481-017-9749-2 28578466

[B43] Pocock J. M. Kettenmann H. (2007). Neurotransmitter receptors on microglia. *Trends Neurosci.* 30 527–535. 10.1016/j.tins.2007.07.007 17904651

[B44] Pruessner J. C. Champagne F. Meaney M. J. Dagher A. (2004). Dopamine release in response to a psychological stress in humans and its relationship to early life maternal care: a positron emission tomography study using [11C] raclopride. *J. Neurosci.* 24 2825–2831. 10.1523/JNEUROSCI.3422-03.2004 15028776 PMC6729514

[B45] Rougé-Pont F. Piazza P. V. Kharouby M. Le Moal M. Simon H. (1993). Higher and longer stress-induced increase in dopamine concentrations in the nucleus accumbens of animals predisposed to amphetamine self-administration. A microdialysis study. *Brain Res.* 602 169–174. 10.1016/0006-8993(93)90260-T 8448654

[B46] Sierra A. Encinas J. M. Deudero J. J. Chancey J. H. Enikolopov G. Overstreet-Wadiche L. S. (2010). Microglia shape adult hippocampal neurogenesis through apoptosis-coupled phagocytosis. *Cell Stem Cell* 7 483–495. 10.1016/j.stem.2010.08.014 20887954 PMC4008496

[B47] Solich J. Faron-Gorecka A. Kusmider M. Palach P. Gaska M. Dziedzicka-Wasylewska M. (2011). Norepinephrine transporter (NET) knock-out upregulates dopamine and serotonin transporters in the mouse brain. *Neurochem. Int.* 59 185–191. 10.1016/j.neuint.2011.04.012 21693154

[B48] Streit W. J. (2002). Microglia as neuroprotective, immunocompetent cells of the CN S. *Glia* 40 133–139. 10.1002/glia.10154 12379901

[B49] Streit W. J. (2004). Microglia and Alzheimer’s disease pathogenesis. *J. Neurosci. Res.* 77 1–8. 10.1002/jnr.20093 15197750

[B50] Streit W. J. Mrak R. E. Griffin W. S. (2004). Microglia and neuroinflammation: a pathological perspective. *J. Neuroinflammation* 1:14. 10.1186/1742-2094-1-14 PMC509427 15285801

[B51] Tanaka K. Okada Y. Kanno T. Otomo A. Yanagisawa Y. Shouguchi-Miyata J. (2008). A dopamine receptor antagonist L-745,870 suppresses microglia activation in spinal cord and mitigates the progression in ALS model mice. *Exp. Neurol.* 211 378–386. 10.1016/j.expneurol.2008.02.004 18423451

[B52] Tanaka T. Ueno M. Yamashita T. (2009). Engulfment of axon debris by microglia requires p38 MAPK activity. *J. Biol. Chem.* 284 21626–21636. 10.1074/jbc.M109.005603 19531468 PMC2755886

[B53] Toda M. Kuo C. H. Borman S. K. Richardson R. M. Inoko A. Inagaki M. (2012). Evidence that formation of vimentin mitogen-activated protein kinase (MAPK) complex mediates mast cell activation following FcepsilonRI/CC chemokine receptor 1 cross-talk. *J. Biol. Chem.* 287 24516–24524. 10.1074/jbc.M111.319624 22613718 PMC3397876

[B54] Troadec J. D. Marien M. Mourlevat S. Debeir T. Ruberg M. Colpaert F. (2002). Activation of the mitogen-activated protein kinase (ERK(1/2)) signaling pathway by cyclic AMP potentiates the neuroprotective effect of the neurotransmitter noradrenaline on dopaminergic neurons. *Mol. Pharmacol.* 62 1043–1052. 10.1124/mol.62.5.1043 12391266

[B55] Tsubouchi A. Sakakura J. Yagi R. Mazaki Y. Schaefer E. Yano H. (2002). Localized suppression of RhoA activity by Tyr31/118-phosphorylated paxillin in cell adhesion and migration. *J. Cell Biol.* 159 673–683. 10.1083/jcb.200202117 12446743 PMC2173105

[B56] Turner C. E. (2000). Paxillin interactions. *J. Cell Sci.* 113(Pt 23), 4139–4140. 11069756 10.1242/jcs.113.23.4139

[B57] Vallone D. Picetti R. Borrelli E. (2000). Structure and function of dopamine receptors. *Neurosci. Biobehav. Rev.* 24 125–132. 10.1016/S0149-7634(99)00063-9 10654668

[B58] Vindis C. Séguélas M. H. Lanier S. Parini A. Cambon C. (2001). Dopamine induces ERK activation in renal epithelial cells through H2O2 produced by monoamine oxidase. *Kidney Int.* 59 76–86. 10.1046/j.1523-1755.2001.00468.x 11135060

[B59] Webb D. J. Donais K. Whitmore L. A. Thomas S. M. Turner C. E. Parsons J. T. (2004). FAK-Src signalling through paxillin, ERK and MLCK regulates adhesion disassembly. *Nat. Cell Biol.* 6 154–161. 10.1038/ncb1094 14743221

[B60] Yan Y. Jiang W. Liu L. Wang X. Ding C. Tian Z. (2015). Dopamine controls systemic inflammation through inhibition of NLRP3 inflammasome. *Cell* 160 62–73. 10.1016/j.cell.2014.11.047 25594175

[B61] Yang F. Lirui X. Chang Y. C. (2017). Signaling pathways controlling microglia chemotaxis. *Mol. Cells* 40 163–168. 10.14348/molcells.2017.0011 28301917 PMC5386953

[B62] Zhao H. Cheng L. Liu Y. Zhang W. Maharjan S. Cui Z. (2014). Mechanisms of anti-inflammatory property of conserved dopamine neurotrophic factor: inhibition of JNK signaling in lipopolysaccharide-induced microglia. *J. Mol. Neurosci.* 52 186–192. 10.1007/s12031-013-0120-7 24078520

[B63] Zhou M. Y. Engel K. Wang J. (2007). Evidence for significant contribution of a newly identified monoamine transporter (PMAT) to serotonin uptake in the human brain. *Biochem. Pharmacol.* 73 147–154. 10.1016/j.bcp.2006.09.008 17046718 PMC1828907

